# Ensemble Learning Framework for DDoS Detection in SDN-Based SCADA Systems

**DOI:** 10.3390/s24010155

**Published:** 2023-12-27

**Authors:** Saadin Oyucu, Onur Polat, Muammer Türkoğlu, Hüseyin Polat, Ahmet Aksöz, Mehmet Tevfik Ağdaş

**Affiliations:** 1 Department of Computer Engineering, Adıyaman University, Adıyaman 02040, Turkey; 2 Department of Computer Engineering, Bingöl University, Bingöl 12000, Turkey; opolat@bingol.edu.tr; 3 Department of Software Engineering, Samsun University, Samsun 55000, Turkey; muammer.turkoglu@samsun.edu.tr; 4 Department of Computer Engineering, Faculty of Technology, Gazi University, Ankara 06500, Turkey; polath@gazi.edu.tr; 5 MOBILERS, Sivas Cumhuriyet University, Sivas 58000, Turkey; aaksoz@cumhuriyet.edu.tr; 6 Department of Computer Technologies, Munzur University, Tunceli 62000, Turkey; mtagdas@munzur.edu.tr

**Keywords:** CPES, renewable energy, SCADA, SDN, smart grids, DDoS attack

## Abstract

Supervisory Control and Data Acquisition (SCADA) systems play a crucial role in overseeing and controlling renewable energy sources like solar, wind, hydro, and geothermal resources. Nevertheless, with the expansion of conventional SCADA network infrastructures, there arise significant challenges in managing and scaling due to increased size, complexity, and device diversity. Using Software Defined Networking (SDN) technology in traditional SCADA network infrastructure offers management, scaling and flexibility benefits. However, as the integration of SDN-based SCADA systems with modern technologies such as the Internet of Things, cloud computing, and big data analytics increases, cybersecurity becomes a major concern for these systems. Therefore, cyber-physical energy systems (CPES) should be considered together with all energy systems. One of the most dangerous types of cyber-attacks against SDN-based SCADA systems is Distributed Denial of Service (DDoS) attacks. DDoS attacks disrupt the management of energy resources, causing service interruptions and increasing operational costs. Therefore, the first step to protect against DDoS attacks in SDN-based SCADA systems is to develop an effective intrusion detection system. This paper proposes a Decision Tree-based Ensemble Learning technique to detect DDoS attacks in SDN-based SCADA systems by accurately distinguishing between normal and DDoS attack traffic. For training and testing the ensemble learning models, normal and DDoS attack traffic data are obtained over a specific simulated experimental network topology. Techniques based on feature selection and hyperparameter tuning are used to optimize the performance of the decision tree ensemble models. Experimental results show that feature selection, combination of different decision tree ensemble models, and hyperparameter tuning can lead to a more accurate machine learning model with better performance detecting DDoS attacks against SDN-based SCADA systems.

## 1. Introduction

Nowadays, the sustainability of renewable energy sources has become increasingly significant. Furthermore, renewable energy sources are quickly gaining popularity as a substitute for fossil fuels. These sources initiate a transition towards a greener energy sector, and the cyber-physical energy system (CPES) is a crucial component for achieving a sustainable energy future. Supervisory Control and Data Acquisition (SCADA) systems are a prominent technology for effectively managing this transformation. Integrating renewable energy and SCADA technologies is crucial in achieving sustainability and future goals in the energy sector. SCADA systems play a critical role in monitoring and managing renewable energy facilities, enhancing efficiency, and ensuring energy security [1]. SCADA systems continuously monitor energy production, the operational status of facilities, efficiency, and performance, rapidly identify potential issues, and provide opportunities for intervention through instant data analysis. Additionally, they ensure that energy production is managed according to demands and environmental conditions [2].

However, traditional SCADA systems suffer from scalability, flexibility, and management issues. Traditional SCADA systems often have a static network structure and have difficulty adapting to rapid changes. This lack of flexibility can become a significant problem when needs and technology constantly change. It can be difficult to quickly integrate different device types and communication protocols and adapt to new requirements [3]. In addition, as the SCADA network infrastructure grows, management and coordination become more complex. Management tasks such as backups, updates, security, and performance optimization can be challenging in large-scale SCADA systems. More advanced technologies and strategies are required to overcome these challenges and manage SCADA systems in a secure, efficient and sustainable manner. Integrating modern technology such as Software Defined Network (SDN) into traditional SCADA systems helps to overcome existing problems. SDN technology allows network resources to be managed with centralized software control differently to traditional network management approaches. Moving to an SDN architecture in traditional SCADA systems provides benefits such as ease of management, scalability, and flexibility [4].

Integrating SCADA systems with modern technologies, such as the Internet of Things (IoT), cloud computing, and big data analytics, offers significant advantages in industrial automation and process control. In addition to applying SDN architecture to traditional SCADA systems, Internet connectivity enables comprehensive monitoring and control over the Internet. This significantly improves traditional SCADA systems, which previously operated in isolated networks. Furthermore, in recent years, cloud computing has been utilized increasingly for data collection, analysis, and management in SCADA systems. Cloud computing is known for storing, processing, and analyzing vast amounts of data. Thus, transferring data from industrial control systems to cloud-based infrastructure and analyzing them can significantly enhance operational efficiency and decision-making processes. Cloud-based SCADA systems move data from industrial plants to the cloud where they are stored, processed, and analyzed using data analytics algorithms. Data analytics aids in decision-making processes by transforming SCADA data into meaningful insights. With data analytics, plant performance can be evaluated, operating processes can be optimized, and potential issues can be identified and resolved in advance [5].

Although integrating advancements in information and communication technologies like the Internet of Things, cloud computing, and big data analytics into SDN-based SCADA systems offers numerous benefits, it also raises concerns regarding cybersecurity [6]. The modernization of the traditional SCADA system, together with standardization of communication protocols, increased interconnectivity of networks, and remote accessibility of systems, has resulted in a surge in cyber-attacks directed at modern SDN-based SCADA systems [7]. Among the methods employed, Distributed Denial of Service (DDoS) attacks are the most perilous. DDoS attacks are on the rise as they require less effort than installing malware in an organization’s IT system. Attackers with little technical expertise can easily execute DDoS attacks with high success rates. Attackers often use infected computers or devices known as botnets to launch attacks. Their aim is to disrupt the normal operations of SCADA systems by overwhelming the network with heavy traffic, thus consuming resources and either disabling or preventing them from functioning as intended. In SDN-based SCADA systems, the central SDN controller is usually the target of such attacks because targeting it proves to be the most effective strategy for attackers. The SDN controller facilitates focusing on a single target to take down the SDN-based SCADA system [8]. The controller manages network resources and communicates with all devices on the network, hence impacting all devices and services on the network when attacked. This enhances the effectiveness of the attack [9]. Overloading the controller can lead to difficulties in managing the system or even cause it to stop functioning [10].

DDoS attacks can disrupt SCADA systems, leading to complete service outages or interruptions. The attack may result in data loss or data corruption, causing the facility to lose historical data, which cannot be analyzed. Additionally, problems in interconnected critical infrastructures that affect each other can trigger larger issues [11]. A DDoS attack on a SCADA system without DDoS attack detection capability can have severe consequences. The SCADA system utilized in renewable energy facilities is vulnerable to DDoS attacks, which can result in the loss of its capacity to control and monitor power generation. Consequently, this can lead to a production outage of renewable energy facilities, ultimately reducing electricity supply. System recovery after an attack can be time-consuming and delay the plant’s return to normal generation capacity. Outages or interruptions to the SCADA system lead to significant economic losses and reputational damage for the business. Therefore, continuously monitoring network traffic and detecting abnormal situations in SDN-based SCADA systems provide early warning against potential attacks [12].

This paper proposes an optimized machine learning technique to detect DDoS attacks in SDN-based SCADA systems by accurately distinguishing between normal and DDoS attack traffic. Specifically, the proposed method utilizes Decision Tree-based Ensemble Learning, which is an ensemble method combining multiple decision trees. This collective approach effectively identifies and distinguishes normal network traffic from DDoS attack traffic. Moreover, the performance of the decision tree ensemble is optimized through feature selection and hyperparameter tuning. This improves the model’s detection of DDoS attacks by effectively processing complex and high-dimensional data. Experimental results demonstrate that the machine learning-based method proposed in this study exhibits high accuracy in detecting DDoS attacks.

The study’s contribution to the literature can be summarized as follows:

Modern SCADA systems have progressed from closed, standalone systems to intricate, advanced, and diverse open systems connected to the Internet. Conventional approaches create complexity when it comes to debugging, optimizing, and configuring new technologies integrated into a complex and diverse system. A new SDN-based SCADA architecture was developed in this study to address the persistent issues plaguing traditional SCADA systems by leveraging the advantages of SDN technology. This approach differs from prior literature in the field.
This study focuses on the security of critical infrastructure systems, specifically DDoS attacks against SCADA systems.
The Decision Tree-based Ensemble Learning algorithm was trained and tested with an original dataset. Feature selection and hyperparameter tuning techniques were used to optimize the algorithm’s performance.
The presented research showcases extensive testing of the proposed methodology, with results revealing high accuracy, sensitivity, and specificity levels. These findings support the practical applicability of the method in real-world scenarios.
The study adds to the existing body of literature on industrial control system cybersecurity by addressing a crucial issue within the field of CPES.

The article provides a detailed analysis of safeguarding SCADA systems against DDoS attacks. The subsequent sections are structured as follows. “Section 2” presents a complete definition of SDN-based SCADA systems, comprehensively elucidating their architectural design, functions, and benefits in managing the challenges posed by contemporary industrial control systems. Next, “Section 3” presents the proposed methodology for effectively detecting and mitigating DDoS attacks in SDN-based SCADA environments. This approach is centered on Decision Tree-based Ensemble Learning. The paper moves on to the Experimental Results section, which presents empirical evidence validating the approach’s effectiveness through high accuracy, sensitivity, and specificity values. Subsequently, the Discussion section presents an objective assessment of the experimental results, leading to definitive conclusions in the Conclusions section. Additionally, the paper concludes with insights into potential future trends, emphasizing avenues for further advancement in safeguarding critical infrastructure systems against ever-evolving cyber threats.

## 2. Related Works

Classical machine learning methods are commonly used to detect DDoS attacks in traditional SCADA systems, as the literature outlines. Diverse classifier techniques, including Support Vector Machine (SVM), Naive Bayes (NB), K-Nearest Neighbor (KNN), Linear Discriminant Analysis (LDA), and Decision Tree (DT), have been employed in prior investigations. Furthermore, a limited number of investigations have utilized deep learning techniques to categorize attacks and employed feature selection and reduction to identify efficient features.

Teixeira, M. A., et al. proposed five traditional machine learning algorithms, namely Random Forest (RF), DT, Logistic Regression (LR), NB, and KNN, to detect attacks on SCADA systems. The study compares the performance obtained during training and testing of machine learning models with the performance obtained during online deployment of these models in the network. The results show that machine learning models are more efficient at detecting attacks in real time [13].

Perez, R. L., et al. assert that current SCADA system security measures are inadequate against attacks. They propose a normalization-based model for predicting missing data in SCADA systems that cannot detect attacks not found in the database. The study employs SVM and RF classifiers for intrusion detection, resulting in a high detection rate for the RF classifier according to experimental results [14].

Manikant Panthi, OneR, NB, SVM, KNN, RF and AdaBoost machine learning algorithms are used to determine whether the power outage in SCADA systems is due to natural causes or a DDoS attack. These algorithms are used to determine whether the network traffic to the system belongs to DDoS attack or normal network traffic. As a result of the results obtained in the study, it is emphasized that it will increase the efficiency of power system design and facilitate the work of operators [15].

Tolgahan Öztürk et al. used a binary classification approach and a classification model with five different attack classes to detect attacks on IoT devices used in SCADA systems. The classifiers used were KNN, SVM and DT. Among these three classifiers, both accuracy and success rate in intrusion detection were obtained when a DT classifier was used [16].

Rajesh, L. and Satyanarayana, P. conducted a study aimed at preventing intrusions in SCADA systems. They achieved feature reduction in the dataset containing normal and attack data by implementing Chi-Square, ANOVA, and LASSO feature-selection methods. The intrusion detection performance was then evaluated using RF, SVM, KNN, and NB machine learning algorithms. In the experimental study of the dataset acquired through feature selection methods, the SVM classifier yielded the highest performance [17].

Islam, U. et al. used multiple classification models to detect DDoS attacks against financial institutions using a banking dataset. SVM, KNN and RF classifiers were used for attack detection. The highest success rate was achieved with the SVM classifier. The study highlighted that classical machine learning algorithms are more successful in attack detection than deep learning approaches [18].

Ahmad, Z. et al. emphasized in their study that existing intrusion detection systems are insufficient to prevent cyber-attacks on SCADA systems. The results of the experimental studies conducted in the study showed that network intrusion detection mechanisms based on ML and DL methods were capable of responding to the problems experienced. In addition, the study compared the performance of deep learning approaches and classical machine learning algorithms in intrusion detection [19].

Saghezchi, F. B. et al. emphasized that industrial systems have become the target of attackers with the integration of new generation information and communication technologies. In particular, Internet of Things (IoT) nodes in industrial systems are vulnerable to attacks. For intrusion detection, 11 different supervised, unsupervised and semi-supervised algorithms were investigated and their performance was compared. The results of the experimental study showed that supervised algorithms outperform both unsupervised and semi-supervised algorithms in intrusion detection [20].

Wang, W. et al. proposed a stacked deep learning method to detect attackers who infiltrate the SCADA system by bypassing IDS-like security systems. They emphasized that the success rate of the proposed method was more successful than machine learning algorithms such as KNN, RF, NB, AdaBoost, SVM, and OneR [21].

A deep learning-based method for detecting early-stage cyber-attacks on electrical networks has been proposed by Presekal et al. Their approach aimed to identify and localize active attack points in Operational Technology networks in real time. The method integrates a hybrid Graph-Convolutional-Long-Term Memory (GC-LSTM) deep learning model and a deep convolutional network specifically tailored for time-series classification-based anomaly detection [22].

Diaba and Elmusrati introduced a hybrid algorithm using a Convolutional Neural Network (CNN) and a Gated Recurrent Unit (GRU) to detect DDoS attacks on microgrids operating with electric vehicles in vehicle-to-grid mode alongside renewable energy sources [23].

Söğüt et al. prepared a scaled-down version of a real water plant using SCADA system as an experimental environment and applied different DDoS attack scenarios to this environment. CNN, LSTM, proposed CNN-LSTM hybrid models, and traditional machine learning models were applied to the data obtained as a result of non-attack and attack scenarios [24].

Mustafa Altaha and Sugwon Hong proposed an unsupervised deep learning-based Function Code Autoencoder IDS (FC-AE-IDS) intrusion detection system for Distributed Network Protocol 3 (DNP3) systems, one of the most widely used protocols in SCADA systems. The main objective is to prevent servers compromised by attackers from evading rule-based packet inspection [25].

Other efficient deep learning and machine learning techniques for detecting DDoS and adversarial attacks on intelligent systems exist. For example, Yang et al. [26] used autoencoder-based systems to detect DDoS attacks. Hussain et al. [27] used autoencoders to detect adversarial attacks on autonomous driving systems. Stocco et al. [28] presented a continuous anomaly detection technique using autoencoders.

In conclusion, the literature on the detection and prevention of cyber-attacks in SCADA systems reveals a prevailing reliance on classical machine learning methodologies, primarily leveraging various classifiers such as SVM, NB, KNN, RF, and DT. While these methods have demonstrated substantial effectiveness in intrusion detection, a limited number of studies have explored the potential of deep learning techniques, highlighting a promising avenue for enhancing detection capabilities. Moreover, recent endeavors have shown the practical application of hybrid models and feature selection methods, underscoring the significance of optimizing models for real-time intrusion detection. Further research in this domain is crucial to explore the synergies between classical machine learning and deep learning methods, enabling robust and efficient protection against evolving cyber threats targeting SCADA systems in critical infrastructure.

### 2.1. Software Defined Network Design

SDN is an innovative network architecture that separates network control from transmission and allows for direct programming. Traditional networking involves switches that use closed systems, with their own control and data planes, supporting manufacturer-specific control interfaces. In contrast, SDN separates the control and data planes, enabling control logic to be transferred to an external device. Switches transform into basic transmission devices. Separating the control and data planes enables the network control and routing functions to be isolated, permits direct programmability of network control, and isolates transmission devices in the data plane from the application and network services. The programmability of the network makes it simpler to add innovations to network management and application development [29].

**Figure 1:**
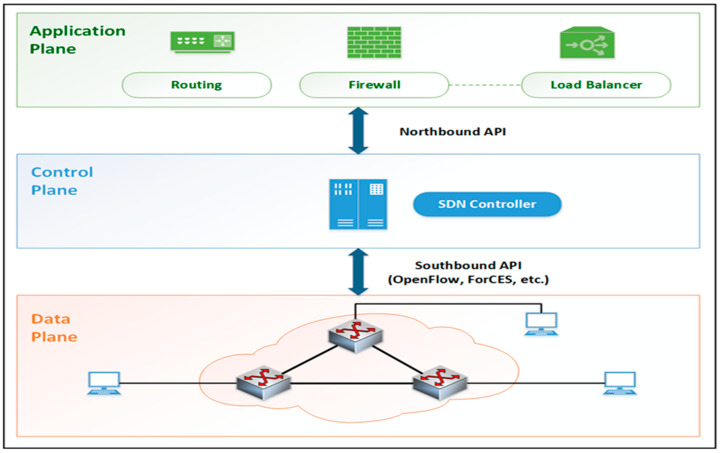
Software Defined Network architecture.

SDN enables the resolution of various limitations present in current network architectures (i.e., operating and hardware costs, network misconfigurations, and related errors) by separating the control and data planes. This transformation shifts static networks towards highly programmable and adaptable ones and offers numerous benefits including robustness, flexibility, performance, usability, scalability, manageability, and security. SDN architecture comprises three primary structures: the application plane, control plane, and data plane (Figure 1).

Data plane: Packet-forwarding components and interfaces, switches, routers, etc. It consists of network components such as routing devices that are connected via wireless radio channels or wired cables. These transmission devices have two main functions. First, they are responsible for collecting network status information (network topology, traffic statistics, etc.), temporarily storing it on local devices, and periodically sending it to the controller. Second, they are responsible for transmitting packets according to the rules set by the controller.

Control plane: The controller, which is the brain of the network, is located in this plane. The controller is responsible for configuring the network and monitoring the devices in the data plane. The controller configures and monitors transmitting devices in the data plane via the southbound interface. This interface facilitates the development and implementation of network services, adding innovation to the network. The OpenFlow protocol is commonly used on the southbound interface. The controller communicates with transmission devices in the data plane through the OpenFlow protocol.

Application plane: Contains SDN applications (routing, firewalls, load balancers, monitoring, etc.) designed to meet user requirements. Due to the programmable platform provided by the control layer, SDN applications can access and control transmission devices located in the data plane. The controller communicates with SDN applications in the application plane through the northbound interface.

### 2.2. SCADA System Definition and Planning

**Figure 2:**
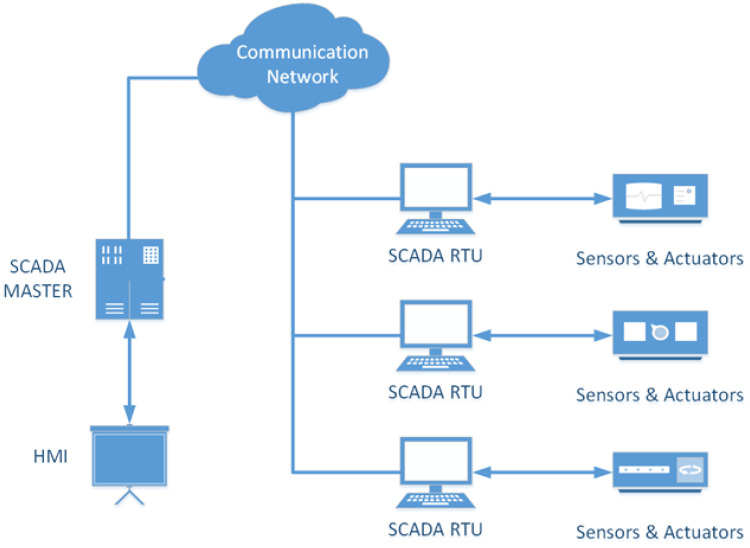
Basic SCADA components.

SCADA systems are used to control and monitor critical infrastructure. These infrastructures include those related to the production and distribution of resources such as water, oil, and gas. SCADA systems have a wide range of applications and serve different sectors. SCADA systems consist of three units: the main terminal unit (MTU), the remote terminal unit (RTU) and the communication network (Figure 2).

The MTU serves as the central monitoring station and is responsible for controlling and commanding the RTU machine via communication links. It also responds to messages from the RTU, processing and storing them for later communication. It is also responsible for collecting data from remote terminals, transmitting these to the Human Machine Interface (HMI), and sending control signals. It also provides the high-level control logic for the system. Communication in this case is carried out using communication protocols specific to SCADA systems, such as Modbus. The RTU exchanges data and commands with the MTU and sends control signals to field devices.

The RTU is responsible for collecting real-time data and information from sensors connected to the physical environment via LAN/WAN connections. RTUs transmit the collected data to the MTU and are also responsible for transmitting the current status data of the physical devices connected to the system. The communication network provides communication services between the various components of the SCADA network framework. The medium used may be wireless or wired. The HMI provides a communication interface between SCADA hardware and software components. It is responsible for controlling operational information in the SCADA system [2].

### 2.3. Distributed Denial of Service

**Figure 3:**
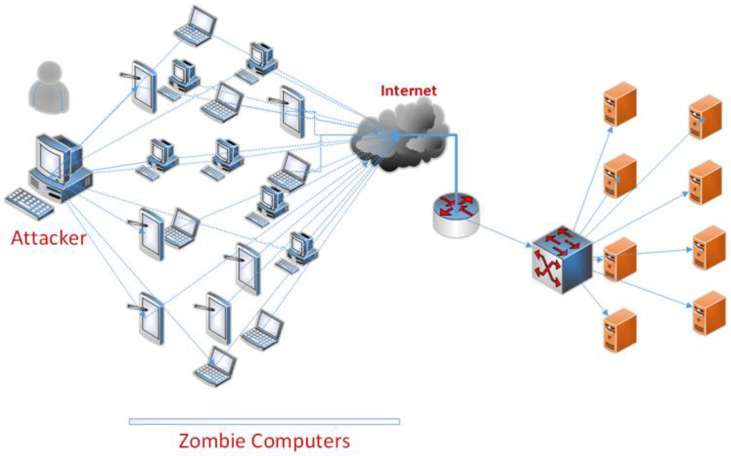
General DDoS attack.

DoS attacks are typically initiated from a solitary computer or resource in an attempt to limit or entirely halt access by overburdening the targeted system or resource. The extent of damage wrought by a DoS attack is dependent upon attacker’s resource strength. A DDoS attack is a type of cyber-attack that seeks to overwhelm the target system by flooding it with traffic from multiple computers or devices. DDoS attacks are typically executed using zombie computers or botnets. The attackers conduct a DDoS attack by directing coordinated traffic through zombie computers to target systems. These attacks can render targeted systems inaccessible by overwhelming resources on a large scale (Figure 3).

During a DDoS attack, the target service and associated services can become inoperable when excessive resources, such as processor, memory, and bandwidth, are consumed. The attacker can easily disguise themselves due to the comprehensive nature of the attack and often employ fake IP addresses, making it difficult to detect the source of the attack.

## 3. Proposed Methodology

**Figure 4:**
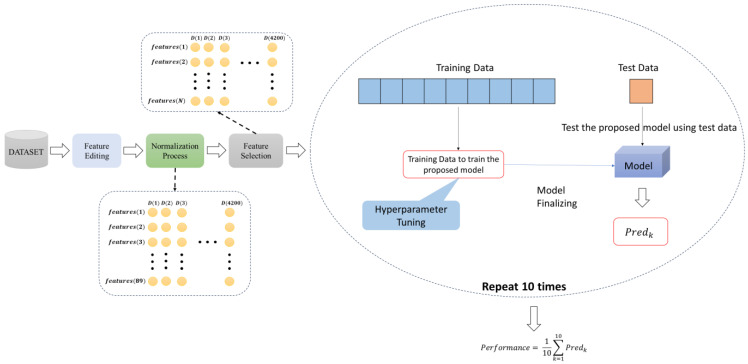
The structure of the proposed methodology.

This paper describes an optimized tree-based ensemble learning method for detecting DDoS attacks in SCADA systems that use SDN. DDoS attacks are known for their intensity and coordination and can cause significant damage to critical infrastructure. To address this issue, we developed a hybrid model that uses machine learning classifier methods to detect DDoS attacks in SDN-based SCADA systems. This proposed model comprises four phases: dataset creation, feature editing, normalization, and classification. Figure 4 provides a visual representation of the proposed system, illustrating all stages.

These stages of the proposed model given in Figure 4 are explained in the sub-sections.

### 3.1. Dataset Generation

**Figure 5:**
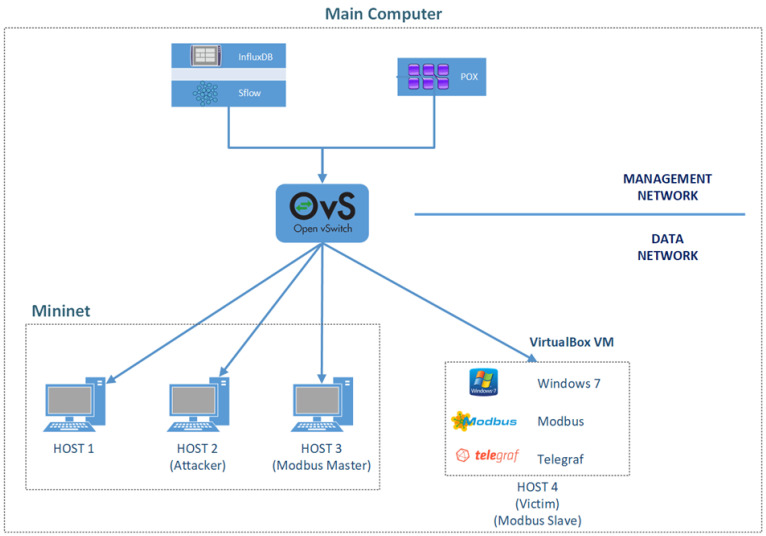
An experimental SDN-based SCADA system was created to collect data.

The experimental topology created to collect DDoS attack data and normal network traffic data for the SDN-based SCADA network is shown in Figure 5. The topology consists of a minimum number of nodes to implement the DDoS attack on the SDN-based SCADA network and to evaluate the effects of the attack. The experimental studies were conducted in a Ubuntu 20.04 LTS operating system on a computer with 32 Gb RAM and an Intel i7-1165g7 processor. In the experimental topology, there are three users named Host 1, Host 2, Host 3, created using Mininet VM/Ubuntu version 2.3.0. In addition, a virtual machine named Host 4 has been created. The Host 4 virtual machine hosts Open vSwitch (OVS) switch and Phyton-based open source OpenFlow/SDN (POX) controller. In addition, sFlow-RT, InfluxDB, and Telegraf applications were installed on Host 4. sFlow was used to collect network data through the OVS switch during and after an attack. The collected network data were stored in the InfluxDB database with a timestamp. With Telegraf application, operating system telemetric values such as CPU, memory, and register values transmitted over Modbus-TCP protocol were collected by sFlow-RT installed on Host4 and the data was stored in InfluxDB database.

Protocol (User Datagram Protocol (UDP), Transmission Control Protocol (TCP) and Internet Control Message Protocol (ICMP) flood) DDoS attacks were generated using the hping3 packet generator tool to collect traffic flow data of the DDoS attack.

The hping3 tool was installed on Host 2 with IP address 10.0.0.0.2 and identified as the attacker, while Host 4 with IP address 10.0.0.10 was selected as the victim. The Modbus protocol uses the master/slave technique to allow communication between users. In the network we have created, the Host 3 computer with the IP address 10.0.0.0.3 is set as the Modbus master, and the Host 4 user is set as the Modbus slave.

The dataset was obtained as a result of a four-step scenario. Each scenario was run within 60 min for each of the TCP, UDP, and ICMP packets sent. During the experimental simulation, these TCP, UDP, and ICMP packets were sent first as normal packets and then as malicious packets. The size of each packet was 512 bytes. The rate of packets sent using hping3 during the attack was over 2000 packets per second. To obtain attack and normal network traffic data from the experimental SDN-based SCADA network, communication was first established between Host 3, the Modbus master node, and Host 4, the Modbus slave. Set register values were received from the slave node to the master node. Then, four-step scenarios were implemented.

- Scenario 1: While Modbus communication is in progress between Host 3, the Modbus master node, and Host 4, the Modbus slave node, a TCP flood attack is performed from user Host 2 to user Host 4.
- Scenario 2: While Modbus communication is in progress between Host 3, the Modbus master node, and Host 4, the Modbus slave node, a UDP flood attack is performed from User Host 2 to User Host 4.
- Scenario 3: While Modbus communication is in progress between Host 3, the Modbus master node, and Host 4, the Modbus slave node, an ICMP flood attack is performed from user Host 2 to user Host 4.
- Scenario 4: While Modbus communication is in progress between Host 3, the Modbus master node, and Host 4, the Modbus slave node, ping packets are sent from the Host 1 user to the Host 4 user to generate normal network traffic.

The features in the obtained dataset contain data specific to the SDN-based SCADA network. As shown in Table 1, the dataset consists of 89 features, 420 normal and 3780 attack data samples [30].

### 3.2. Pre-Processing

This study concentrated on efficiently detecting DDoS attacks in SDN-based SCADA systems. The preparation of the dataset proved crucial to accomplishing this aim. Initially, we gathered network traffic data as a direct outcome of the scenarios implemented in the experimental topology. Pre-processing was subsequently utilized to convert these data into a practical dataset. At this stage, we ensured that the data were formatted consistently and any unnecessary duplicates were removed. Addressing missing data was also paramount, as it can have adverse effects on the analysis and modeling processes. Therefore, a careful approach was taken when handling missing data. In most cases, the missing values of relevant features were successfully filled by averaging these features. This ensured data integrity and contributed to more reliable results. This data pre-processing aimed to prepare the dataset for both model training and testing. It was a crucial step towards developing an effective DDoS attack detection model. Through this preparation, we were able to perform a reliable analysis and increase the overall result accuracy.

### 3.3. Feature Selection

Minimum Redundancy Maximum Relevance (MrMR) is a feature selection approach and is mainly used in machine learning and data mining [31]. MrMR is a feature selection method that aims to balance the features in a dataset. This method aims to ensure that the selected features have minimum redundancies with each other and at the same time have maximum relevance to the target variable (label) [32].

Assume that there are “n” features and “m” instances in the dataset. Let the features be $X_{1},X_{2}\ldots .,X_{n}$ and the target variable Y.

- Minimum Redundancy: MrMR aims to minimize the similarity between selected features. This can be carried out by measuring the correlation between two features [33]. Correlation can be measured using Pearson’s correlation coefficient [34]: Pearson Correlation Coefficient

(1) $$\left(r\right)=(\sum \left(\left(X_{i}-\mu_{X}\right)\left(X_{j}-\mu_{Y}\right))\right)/(\sigma_{X}*\sigma_{Y})$$

Here, *i* and *j* represent the features, $\mu_{X}$ and $\mu_{Y}$ represent the means of the features, and $\sigma_{X}$ and $\sigma_{Y})$ represent the standard deviations. This coefficient measures the strength of the relationship between two features. The MrMR method attempts to minimize this correlation.

- Maximum Relevance: MrMR aims to ensure that the selected features have maximum relevance to the target variable. This can be achieved by measuring the relationship between the features and the target variable objectively. For instance, statistical tests like *t*-test or ANOVA may be employed. To measure the significance of the relationship between two features using a *t*-test, the following equation can be utilized [31]:

(2) $$t=(\mu_{1}-\mu_{2})/sqrt(\left({\sigma_{1}}^{2}/n_{1}\right)+\left({\sigma_{2}}^{2}/n_{2}\right))$$

Here, $\mu_{1}$ and $\mu_{2}$ represent the means of the two features concerning the target variable, and $\sigma_{1}$ and $\sigma_{2}$ represent their standard deviations. $n_{1}$ and $n_{2}$ show the sample numbers for both groups. MrMR tries to maximize this *t* value [34].

The MrMR method combines the principles of minimizing repetition and maximizing relevance in a balanced manner. The ideal selected features should have minimum similarity to each other while being highly relevant to the target variable. Therefore, MrMR feature selection aims to reduce feature similarity in the dataset while maximizing their relevance to the target variable. This leads to a more efficient subset with fewer features, resulting in improved outcomes for predictors or classifiers [35].

### 3.4. Ensemble Learning

The model proposed in this paper uses ensemble methods that combine decision trees to achieve better prediction performance than using a single decision tree. The basic principle of this ensemble model is that a collection of weak learners can form a strong learner. This method builds a large number of decision trees using different subsets of the data or different features [36]. Ensemble models combine the predictions of each decision tree to create a stronger and more stable predictor. This allows the model to make more general and reliable predictions. In addition, ensemble methods can help compensate for errors made by a single tree [37]. In the current study, Boosting, Bagging (Bootstrap Aggregating) and Random Under-Sampling Boosting (RUSBoost) techniques are used. Each of them uses different strategies to combine decision trees and thus improve the learning process. In conclusion, ensemble methods are a powerful tool to maximize the potential of weak learners such as decision trees and improve prediction performance.

These methods are briefly detailed in the items:

- Decision Tree-based Ensemble Boosting Method: Boosting is an ensemble learning approach that builds a strong classifier using basic learners called weak learners. First, it starts with an initial weak learner (usually a decision tree) and identifies mispredicted instances of the dataset. Focusing on these instances, the next weak learner is trained and the process is repeated. Each weak learner is heavily weighted to correct the errors of the previous learners. As a result, these combined weak learners form a strong learner that can make stronger and more accurate predictions in situations where it might have failed on its own. Boosted Trees classification is known for its ability to provide high performance and accuracy in classification problems and is often used successfully in real-world applications [38].
- Decision Tree-based Ensemble Bagging Method: The basic idea of bagging is to train different models on random subsets of data so that each model learns from a different perspective, and then aggregates them to make a stronger prediction. Bagging is particularly effective at reducing variance and avoiding overfitting. The key to the method is how each sample of the dataset is prepared to train the ensemble base models. Bagging uses a random sampling method called bootstrap sampling. Random samples are taken from the dataset, and these samples are used to create different sub-datasets. This process involves repeatedly creating datasets with randomly selected samples from the original dataset. The number of subsamples generated is equal to the number of samples in the original dataset. For this reason, some samples may not be included in the samples generated as a result of the bootstrap, while others may appear two or more times. After the training dataset is created, any samples not included in the training dataset are transferred to the test dataset. For each sub-dataset, base models are created using the same or a different machine learning model. Each base model is trained with its own bootstrap data subset. Each base model can use the same algorithm or different algorithms. The models run in parallel and are independent of each other. The final predictions are determined by combining the predictions of all the models. The base models make their predictions, which are usually combined by voting in classification problems or averaging in regression problems.
- The Decision Tree-based Ensemble RUSBoost Method: RUSBoost is an ensemble learning method designed explicitly for unbalanced class problems. It combines traditional boosting methods with a sampling strategy called RUSBoost. With RUSBoost, the majority of class instances are undersampled to reduce imbalance and create a more balanced dataset. Boosting is then applied to the balanced dataset using a weighted combination of weak learners to produce a robust classifier. RUSBoost Trees is a variant of this method that uses decision trees as weak learners. They build a decision tree-based ensemble using different subsets of the training set, and each tree is built on a random undersampled subset of the training data. By combining multiple decision trees, the ensemble model improves the quality of predictions and creates a more reliable classifier. RUSBoost Trees effectively addresses the problem of class imbalance and could be a promising approach in many applications where the minority class is of interest.

This study aims to efficiently detect DDoS attacks utilizing a Decision Tree-based Ensemble Learning approach for optimal performance.

## 4. Experimental Results

The training and testing of the ensemble learning models in this study were performed on the MATLAB platform. The 10-fold cross-validation method was used to test the models trained on the dataset. The following metrics were used to evaluate the performance results of the models:

(3) $$Accuracy=\frac{True\;Positives}{Total\;data}$$

(4) $$Precision=\frac{True\;Positives}{True\;Positives\;+\;False\;Positives}$$

(5) $$Recall=\frac{True\;Positives}{True\;Positives\;+\;False\;Negatives}$$

(6) $$F1-Score=\frac{2\times Precision\times Recall}{Precision+Recall}$$

These metrics are commonly used to evaluate the performance of a classification model. While accuracy measures overall model performance, sensitivity and specificity provide more specific information, and the F1 score balances these two metrics.

In this study, experimental studies were conducted for four different models.

**Figure 6:**
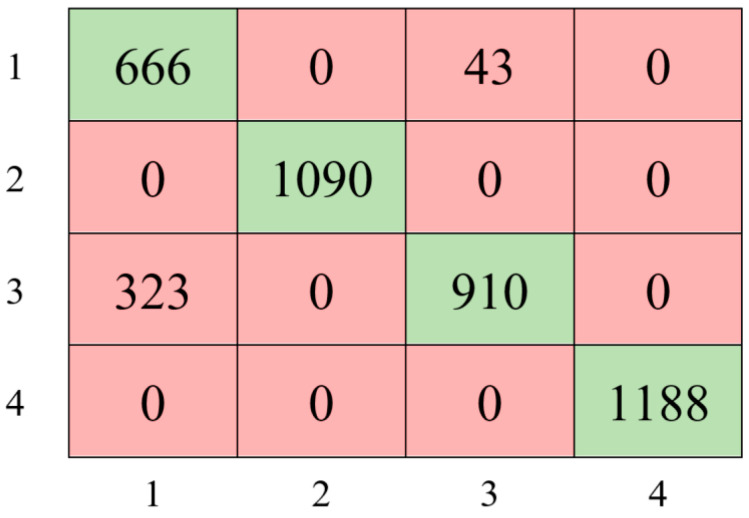
The confusion matrix with the Decision Tree classifier.

The first model was built with a decision tree using pre-processed data. In the training of this first decision tree model, the classifier type, the maximum number of splits, and the split criterion parameters were set to Coarse Tree, 4, and Gini’s diversity index, respectively. The confusion matrix obtained according to the test results of the decision tree model is shown in Figure 6.

The Decision Tree classifier achieved an accuracy of 91.33%, precision of 91.94%, recall of 90.71%, and an F1 score value of 91.32%.

The second model is built using Decision Tree-based Ensemble Boosting method. In the training of this model, learner type, maximum number of splits, number of learners, and learning rate were set to AdaBoost, Decision Tree, 20, 30 and 0.1, respectively.

The third model is built using Decision Tree-based Ensemble Bagging method. In the training of this model, learner type, maximum number of splits, and number of learners were set to Bag, Decision Tree, 4219, and 30, respectively.

The fourth model is built using the Decision Tree-based Ensemble RUSBoost method. In the training of this model, learner type, maximum number of splits, number of learners, and learning rate were set to RUSBoost, Decision Tree, 20, 30 and 0.1, respectively.

The performance results obtained based on decision tree and ensemble models are given in Table 2.

As shown in Table 2, the highest accuracy performance was 92.9% for the model created with the Ensemble Boosted Trees method. However, all the methods based on the ensemble approach produced accuracy values close to each other and provided accuracy values above 92.5%. In addition, it was observed that the models built using the ensemble approach achieved a performance improvement of 1% compared to the Decision Tree classifier model.

To further improve the performance of ensemble learning models, the next experimental study aimed to optimize the parameters of decision tree-based ensemble classifier models. To achieve this goal, we used Bayesian optimization, which is an effective method when the hyperparameters are complex or have a large range. The basic principle of Bayesian optimization is to optimize the objective function by evaluating different combinations in the hyperparameter space. This method is faster and uses less computational resources than trial and error. Bayesian optimization has helped to achieve efficient and successful results in hyperparameter optimization. The parameters to be optimized in Bayesian optimization are shown in Table 3.

**Figure 7:**
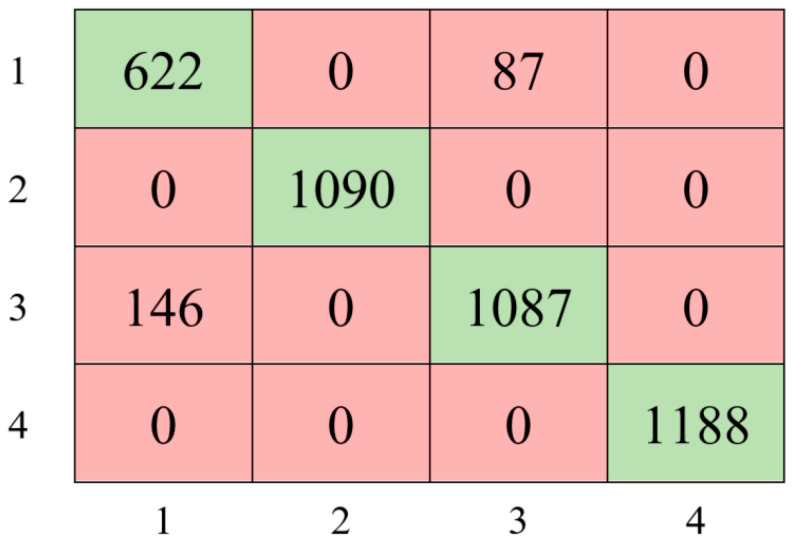
The confusion matrix of the optimized ensemble classifier.

The experimental study utilizing the Bayesian optimization method resulted in achieving an accuracy of 94.48% with the following parameter values: AdaBoost, 21 for maximum number of splits, 11 for number of learners, and 0.0012041 for learning rate. Based on these findings, Figure 7 presents the confusion matrix that illustrates the AdaBoost-based model’s classification performance.

As shown in Figure 7, the model achieved 100% accuracy in classifying classes 2 and 4, while classes 1 and 3 were classified with 87.73% and 88.16% accuracy, respectively. Furthermore, the hyperparameter optimization-based AdaBoost ensemble approach improved the model’s performance by 3% compared to the Decision Tree classifier model.

**Figure 8:**
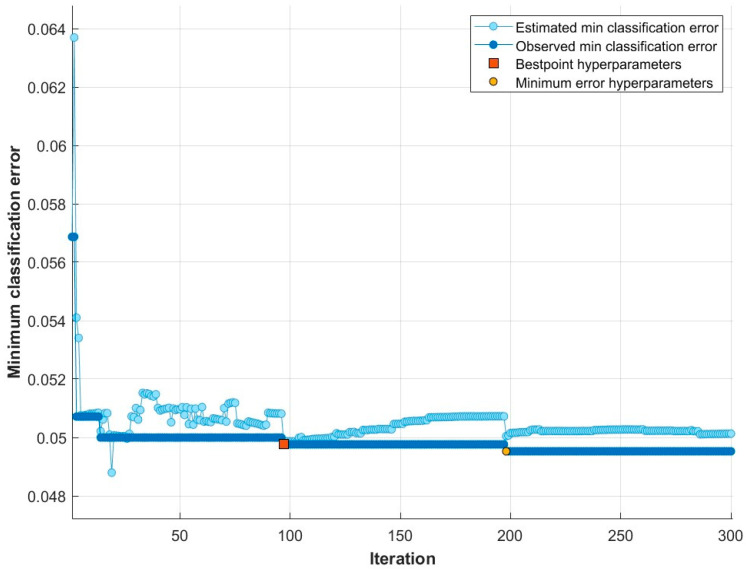
Minimum classification error plot of Bayesian optimization-based proposed ensemble learning classifier.

In the experiment based on hyperparameter optimization, we utilized the MrMR feature selection algorithm to select the dataset’s most significant and efficient features. We computed the score values of each feature and ranked them in accordance with these scores. For each ranked feature set, we utilized the Bayesian optimization algorithm based on the parameter ranges outlined in Table 3 to establish the classifier parameters. As a result of this experiment, Figure 8 displays the graph of the minimum classification error for the ensemble learning approach proposed based on Bayesian optimization.

**Figure 9:**
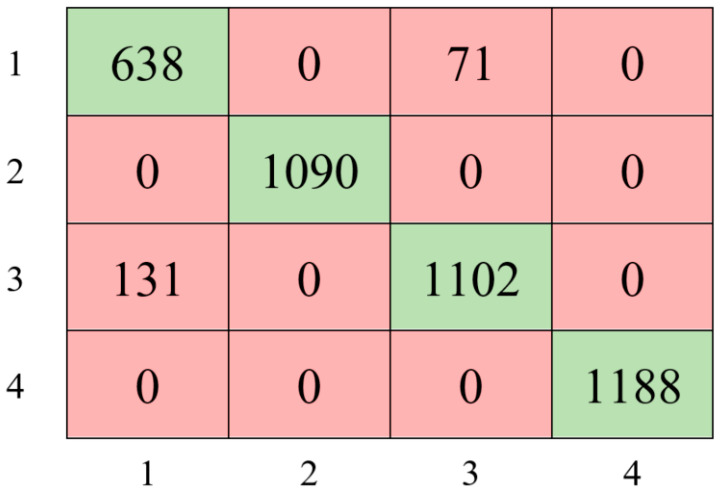
The confusion matrix of the optimized ensemble classifier is based on selected features.

The Bayesian optimization process, using the ensemble learning classifier, was conducted over 30 iterations as shown in Figure 8. At the end of the 14th iteration, the best hyperparameter was obtained. Consequently, a peak performance of 95.17% was achieved with 45 features by utilizing AdaBoost, 12 Maximum number of splits, 18 Number of learners and 0.30254 Learning rate parameter values. The classification performance’s confusion matrix based on this result is displayed in Figure 9.

Based on the confusion matrix performance results presented in Figure 9, feature selection improved model performance by approximately 1% compared to the raw data, allowing for higher accuracy with fewer features. Narrowing the dataset to meaningful and influential features through feature selection resulted in more precise and efficient predictions. The results emphasize the significance of the feature selection process and its potential to enhance performance in applications of machine learning.

## 5. Discussion

The energy sector is undergoing a significant transformation in environmental sustainability and energy efficiency. Renewable energy sources are an essential part of the energy generation portfolio in this transformation. SCADA systems are critical in effectively monitoring and managing renewable energy sources. However, when SCADA systems are built on traditional network architectures, they need more flexibility in network scaling and traffic management. SDN-based SCADA systems are being developed to overcome these limitations instead of traditional SCADA systems. Unlike traditional network management, SDN technology can increase operational efficiency even in complex infrastructures with its programmable dynamic structure. SCADA systems can become more efficient and adaptable in terms of management and scalability by moving to SDN architecture. However, the increasing integration of SDN-based SCADA systems with modern technologies such as the Internet of Things, cloud computing, and big data analytics raises some cybersecurity concerns. A grave cybersecurity threat, DDoS attacks can disrupt the management of energy resources, resulting in service interruptions and increased operational costs. Therefore, it is vital to be prepared for DDoS attacks. The first step in preparing for DDoS attacks is to detect them effectively. Research is currently focused on machine learning-based methods to detect DDoS attacks. These machine learning-based methods can detect attacks by distinguishing between normal and abnormal traffic. In this paper, we propose a method to identify DDoS attacks by applying an optimized decision tree-based ensemble learning approach. This approach is based on an ensemble method combining multiple decision trees. In the present study, decision tree-based ensemble methods such as Boosting, Bagging and RUSBoost are used. These methods use different tactics to merge decision trees and thus improve the learning process. Furthermore, techniques based on feature selection and hyperparameter tuning are used to optimize the performance of the decision tree ensemble. This enhances the machine learning model’s ability to detect DDoS attacks by efficiently processing high-dimensional and complex data.

The results of this study will make a valuable contribution to the development of reliable and secure SCADA infrastructures in the energy sector by further promoting the integration of SDN technology into SCADA systems and security solutions in the future. There are some published works in the literature to address this problem. However, these studies usually use anonymized datasets obtained from traditional SCADA systems or anonymous datasets for machine learning-based DDoS attack detection. However, in this study, a unique dataset obtained from a simulation of an SDN-based SCADA system is used. Table 4 compares the method proposed in this paper with some existing works in the literature in terms of datasets used, machine learning methods, and model accuracy.

As can be seen from the results presented in Table 4, DDoS attack detection based on traditional SCADA systems has generally achieved accuracy rates of 95% and above. In these studies, high performances were generally achieved based on machine learning classifier methods. However, in this study, a unique dataset was created using a real-time SDN-based SCADA system and this dataset has a more challenging structure than other SCADA datasets. For this reason, it is more difficult to achieve performance levels of 99% and above over SCADA. As a result, it would not be a correct approach to fairly compare the proposed model with other studies since different datasets are used. This study differs from most studies in the existing literature by using a unique dataset and a real-time SCADA system in a more challenging test environment. Therefore, the performance results obtained should be considered as a general reference, considering the specific dataset and system conditions.

## 6. Conclusions and Future Trends

This study emphasizes that SCADA systems are gaining importance with the increase in renewable energy sources. However, the size, complexity and management difficulties of SCADA systems require the use of SDN technology. It is stated that SDN-based SCADA systems have cybersecurity concerns and that DDoS attacks pose a threat in particular. Therefore, the study aims to develop an effective detection system against DDoS attacks in SDN-based SCADA systems.

The proposed Ensemble Learning approach was used to distinguish between normal network traffic and DDoS attack traffic. This method is trained and tested on an experimental network topology-based dataset. Techniques such as feature selection and hyperparameter tuning have been applied to optimize the performance of the decision tree ensemble. The reliability of the study was assessed using 10-fold cross-validation and confirmed that the generalization ability of the method was robust. The experimental results obtained show that the proposed model is achieved with high accuracy, with an accuracy rate of 95.2%, a sensitivity rate of 97.3%, and a specificity rate of 94.8%. These results show that this method can provide a more performant and sensitive machine learning model for the detection of DDoS attacks in SDN-based SCADA systems. This could be an important step towards improving security in the energy sector. In conclusion, this study demonstrates the usability of an optimized Decision Tree-based Ensemble Learning approach to increase the security of SCADA systems and provide more effective protection against DDoS attacks.

Future work may focus on further improving this method and extending it against different threats. Additionally, further research can be carried out testing it in real-world applications and applicability on an industrial scale. This study has the potential to present a new paradigm in the field of cybersecurity and can serve as a basis for future research.

## Figures and Tables

**Table 1:** Attributes in the dataset.

Available_Byteswin_mem	Bytes_Received_persecwin_net	Bytes_Sent_persecwin_net
Cache_Faults_persecwin_mem	Context_Switches_persecwin_system	Current_Disk_Queue_Lengthwin_disk
currentmodbus	Demand_Zero_Faults_persecwin_mem	energymodbus
frequencymodbus	packets_received_persecwin	packets_sent_persecwin
page_faults_persecwin	pages_persecwin_mem	percent_Disk_Read_Timewin_diskreversevm
percent_disk_timewin_disk	percent_disk_write_timewin_di	percent_idle_timewin_cpu
percent_idle_timewin_disk	percent_interrupt_timewin_cpu	percent_privileged_timewin_cpu
percent_processor_timewin_cpu	percent_user_timewin_cpu	pool_nonpaged_byteswin_mem
pool_paged_byteswin_mem	power_factormodbus	powermod
powerfactor	processor_queue_lengthwin_system	analyzer_agent_count
analyzer_cpu_available_cores	analyzer_cpu_load_process	analyzer_cpu_load_system
analyzer_cpu_time_ms	analyzer_discarded_datagrams	analyzer_gc_count
analyzer_gc_time_ms	analyzer_heap_max_bytes	analyzer_heap_used_bytes
analyzer_http_connections_current	analyzer_http_connections_total	analyzer_http_received_bytes
analyzer_http_received_messages	analyzer_http_sent_bytes	analyzer_http_sent_messages
analyzer_mem_free_bytes	analyzer_mem_total_bytes	analyzer_parse_errors
analyzer_received_bytes	analyzer_received_datagrams	analyzer_unsupported_version_datagrams
analyzer_uptime_ms	app_conn_max	app_conn_open
app_fd_max	app_fd_open	app_mem_max
app_mem_used	app_systemtime	app_usertime
eth_alignmenterrors	eth_fcserrors	ifindiscards
ifinerrors	ifinmulticastpkts	ifinoctets
ifinpkts	ifinucastpkts	ifinutilization
ifoutdiscards	ifouterrors	ifoutoctets
ifoutpkts	ifoutucastpkts	ifoututilization
ifspeed	ovs_dp_flows	ovs_dp_hitrate
ovs_dp_hits	ovs_dp_lost	ovs_dp_maskhitsperpacket
ovs_dp_masks	ovs_dp_misses	ovs_dp_missrate
ovs_mask_hits	System_Calls_persecwin_system	Transition_Faults_persecwin_memreversevmMemory
voltagemodbus	usage_idle_cpu_total	

Class: Traffic class. The data in the dataset used in this study are labeled data. These four classes; are “Normal”, “ICMP Flood”, “TCP Flood” and “UDP Flood”.

**Table 2:** Performance results of the experimental study.

	Accuracy	Precision	Recall	F1-Score
Decision Tree	91.33	91.94	90.71	91.32
Ensemble Boosted Trees	92.9	93.22	91.96	92.58
Ensemble Bagged Trees	92.7	90.66	92.01	91.32
Ensemble RUSBoost Trees	92.6	92.99	91.71	92.35

**Table 3:** Optimized parameters.

Ensemble Method	AdaBoost, RUSBoost, Bag
Maximum number of splits	1–4219
Number of learners	0.001–1
Learning rate	10–500

**Table 4:** Comparison of previous studies in the literature.

Ref.	Datasets	ML Algorithms	Accuracy (%)
[13]	Their own dataset	RF	99.89
		KNN	72.29
[14]	[7]	SVM	Average 92
		RF	99.4
[15]	Industrial Control System (ICS) Cyber Attack datasets	RF + AdaBoost	Average 90
[16]	[39]	KNN	Average 91
		SVM	Average 79
		DT	98.8
[17]	Their own dataset	RF	Average 99
		SVM	
		KNN	
		NB	
[18]	Banking Fraud Detection	KNN	97.5
		SVM	99.5
		RF	98.7
[22]	Their own dataset	GC-LSTM	96
[24]	Their own dataset	CNN-LSTM	94.73
[25]	Their own dataset	Autoencoder	95
[40]	Their own dataset	KNN	Average 99.63
		SVM	Average 93.81
[41]	Their own dataset from traditional network architecture	Fuzzy Logic-Based Classifier	94.2
[42]	SDN-specific dataset CICDDoS2019 dataset	Stacking Ensemble Architecture	99.6 ~100
[43]	Their own dataset from traditional network architecture	Cost-Sensitive Gradient Boosting DT	98.0
[44]	NSL-KDD dataset	Hierarchical Bayesian Network	98.4
[45]	Their own dataset from traditional network architecture	CNN	91.0
[46]	Their own dataset from SDN architecture	Stacked Autoencoder (SAE)	95.65
[47]	Their own dataset from SDN architecture	NB, DT, SVM	Average 97.2
Our Study	Our dataset	Decision Tree-based Ensemble Learning	95.2

## Data Availability

Data are contained within the article.
